# Perspectives of Turkish family physicians in Istanbul about e-cigarettes as smoking cessation aids: a cross-sectional survey study

**DOI:** 10.1186/s13722-026-00707-w

**Published:** 2026-07-27

**Authors:** Melis Selamoglu, Ayse Emel Onal, Meryem Merve Oren, Selma Karabey, Mahmut Talha Ucar, Sowmya Malamardi, Chris Barton

**Affiliations:** 1 Department of General Practice, School of Public Health and Preventive Medicine, Monash University, 553 St Kilda Rd, Level 5, Melbourne, VIC 3181 Australia; 2 Department of Public Health, Faculty of Medicine, Istanbul University, Istanbul, Türkiye; 3 Saray District Health Directorate, Tekirdağ Provincial Health Directorate, Ministry of Health, Tekirdağ, Türkiye; 4 Department of Public Health, Hamidiye Faculty of Medicine, University of Health Sciences, Istanbul, Türkiye; 5 Department of Public Health, School of Psychology and Public Health, La Trobe University, Melbourne, Australia; 6 Department of General Practice, School of Public Health and Preventive Medicine Monash University, Melbourne, Australia

**Keywords:** E-cigarette, Smoking Cessation, Public Health, Family Physician, Primary Care, General Practice, Healthcare

## Abstract

**Background:**

There has been limited research among family physicians (FPs) in Türkiye regarding smoking cessation and few studies have examined their perceptions of e-cigarettes as smoking cessation aids. This study aimed to examine the knowledge, attitudes and beliefs of FPs in Istanbul about e-cigarettes and their potential role in smoking cessation.

**Methods:**

A cross-sectional online survey was distributed via email to a convenience sample of FPs in Istanbul. Participants were recruited from a list of family practice clinics provided by the Istanbul Provincial Health Directorate and other social media platforms. Data was collected from August until October 2022. Survey items were drawn from published literature and informed by the Theory of Planned Behaviour. Items were translated to Turkish and assessed knowledge, attitudes, beliefs, and willingness to recommend e-cigarettes as a smoking cessation aid to patients who had failed to quit using other methods.

**Results:**

We received 243 responses from male (*n* = 116, 47.7%) and female (*n* = 127, 52.3%) FPs with an average age of 42 ± 10 years and an average of 9 ± 6 years clinical experience. Most participants believed it was their responsibility to aid patients in getting the correct help to quit smoking (*n* = 159/217, 73.3%) and that current medications are effective in helping patients quit smoking (*n* = 171/217, 78.8%). Most disagreed that e-cigarettes were suitable as smoking cessation aids (*n* = 169/217, 77.9%) or safer than regular cigarettes (*n* = 165/214, 77.1%). Respondents were concerned e-cigarettes were a gateway to smoking (*n* = 153/217, 70.5%), addictive (*n* = 166/217, 76.5%) and harmful to the health of the user (*n* = 162/215, 75.3%). Over half stated they were not confident in their level of knowledge about e-cigarettes (*n* = 126/200, 63%), or their ability to answer patient questions (*n* = 119/200, 59.5%) and talk to patients about e-cigarettes (*n* = 121/199, 60.8%). More than half stated they would not recommend e-cigarettes to patients that refuse to take alternative treatments (*n* = 121/197, 61.4%) or to patients that have failed to quit using other methods (*n* = 118/197, 59.9%).

**Conclusion:**

Family physicians in Istanbul demonstrated poor knowledge and confidence regarding e-cigarettes and generally held negative attitudes toward their use for smoking cessation. Knowledge and beliefs about effectiveness of e-cigarettes were key factors associated with recommendation practices. These findings highlight the need for education and guidance to support FPs in responding to patient enquiries about e-cigarettes, in line with the current regulatory context in Türkiye.

**Supplementary Information:**

The online version contains supplementary material available at 10.1186/s13722-026-00707-w.

## Introduction

Türkiye became one of the first nations to ratify the World Health Organization Framework Convention on Tobacco Control (FCTC) in 2004 and implemented a range of significant tobacco control policies to reduce the rate of smoking after signing the FCTC [1]. Law 4207 (Prevention of Harmful Effects of Tobacco Products) was amended in 2008 to introduce various tobacco control policies which included smoke-free policies in public indoor areas (hospitals/clinics, educational buildings, sporting and cultural facilities), tax increases on tobacco products, bans on promotional and advertising materials, inclusion of pictorial health warning labels on cigarette packages and most recently in 2020, implementing tobacco plain packaging policy [1, 2]. Following the amendment of Law 4207, the Global Adult Tobacco Survey reported daily smoking prevalence among adults aged 15 years and over declining substantially, falling from 31.2% in 2008 to 27.1% in 2012 [1, 3]. According to the Turkish Statistical Institute, the 2025 Turkish Health Survey reported that 30.1% of individuals aged 15 years and over were smoking daily [4]. Together, these findings indicate that although daily smoking prevalence declined following the amendment of Law 4207, it has since increased again in 2025.

Smoking cessation clinics in Türkiye are only available within selected hospital and primary care settings and were established in the early 1990’s [5, 6]. In 2025, 469 smoking cessation clinics around the country reported to be providing these services [2]. The Smoking Cessation Treatment Support Program was developed by the Ministry of Health to provide free treatments to individuals who register with these clinics [5]. Each clinic typically includes a trained physician to deliver smoking cessation treatment, psychologist, and supporting health staff to deliver smoking cessation interventions [5, 6].

Electronic cigarettes (e-cigarettes) are battery powered products that heat liquids (e-liquids) to produce an aerosol which is more commonly known as vaping and are available in two formats, open devices which are refillable and closed devices which are disposable [7]. They do not contain tobacco however they do contain propylene glycol, glycerine, flavouring agents and other toxic chemicals that may be harmful to the health of the user [8, 9]. Nearly all e-cigarette liquids contain some level of nicotine, although limited regulation about labelling of these devices means that this is not always made clear to consumers [10].

In the past decade e-cigarette use has increased among young adults and teenagers internationally [11–14]. Concerns over rising levels of nicotine addiction and growing understanding of potential harms from vaping have led to a range of policy and health promotion action to address youth vaping. People who do not smoke and younger individuals are particularly susceptible to the harmful effects of e-cigarettes, facing elevated risks like addiction, which is harmful to the developing brain among younger adults, poisoning from nicotine and other harmful chemicals, inhalation related toxicity, and a higher likelihood of initiating smoking [13]. Despite multiple harmful effects of e-cigarette use, e-cigarettes have also been promoted as smoking cessation aids and there has been interest in their use to support smoking cessation in a number of countries [15].

In Türkiye, the sale, marketing, advertisement and use of e-cigarettes indoors is prohibited, and they are not approved for use as smoking cessation aids by the Turkish Ministry of Health [16–18]. Despite restrictions on the sale and use of e-cigarettes in Türkiye, individuals are able to purchase these products online or import them into the country from other nations [16, 17]. There is no current official national data on the prevalence of e-cigarette use in Türkiye, however, according to Cakir (2023), the prevalence of daily and occasional e-cigarette use among individuals aged 15 years and over is 0.4% [19], although these data were collected in 2019 prior to rapid increase in prevalence of vaping that occurred in many countries through the 2020’s [20, 21]. Authorities and healthcare physicians in Türkiye voice their concerns about e-cigarettes being harmful, addictive, causing various health conditions such as e-cigarette or vaping use-associated lung injury, chronic obstructive pulmonary disease, asthma and lung cancer, having negative effects on the developing brains of teenagers and being a gateway to smoking and other tobacco products [16, 22–24].

A recent cross-sectional study conducted among family physicians in Istanbul examined awareness, perceptions, and clinical practices related to e-cigarettes, with a particular focus on their use as a harm reduction strategy [16]. This study found that only a small proportion of family physicians reported recommending e-cigarettes to patients, and that overall confidence in providing smoking cessation counselling was limited [16]. In addition, physicians’ recommendations were associated with their beliefs about the relative safety and effectiveness of e-cigarettes [16]. While these findings provide important insights into physician perceptions and practices within the Turkish context, the relationships between physicians’ knowledge, attitudes, and perceived competence were not examined within an explicit theoretical framework. As a result, the mechanisms underlying physicians’ intentions and clinical decision-making remain unclear.

Family physicians are key providers of preventive care and play a central role in supporting smoking cessation, including responding to patient questions about emerging nicotine products. In this context, understanding how knowledge, attitudes, and perceived behavioural control shape physicians’ responses to patient enquiries is important for informing clinical practice. Therefore, this study aimed to examine the knowledge, attitudes, and perceived behavioural control of family physicians in Istanbul regarding e-cigarettes, and to assess how these factors are associated with providing advice to patients who may enquire about e-cigarettes for smoking cessation.

Guided by the Theory of Planned Behaviour (TPB) [25], this study explores how knowledge may inform attitudes and perceived behavioural control, and how these constructs are associated with physicians’ intentions regarding the recommendation of e-cigarettes in clinical discussions.

## Methods

### Participants and sampling

A cross-sectional survey was conducted with family physicians practicing in Istanbul, Türkiye, between August and October 2022. Family physicians from 39 districts in Istanbul were invited to complete an online self-administered electronic survey created using Qualtrics. Eligible participants were physicians currently working in family practice settings within Istanbul at the time of the study.

A convenience sampling approach was used. Participants were recruited through two primary channels. First, the survey was distributed to family practice clinics via a list of clinics provided by the Istanbul Provincial Health Directorate. Second, dissemination through professional networks using social media platforms (e.g. WhatsApp^™^) by study investigators (MS, AEO, MTU). The survey link was distributed with clinic contacts and professional groups, who were invited to circulate the survey among eligible physicians.

Due to the use of multiple dissemination pathways and the absence of a centralised registry of physicians contacted, the total number of physicians who received the survey invitation could not be determined, and therefore a response rate could not be calculated. Reminder messages were not systematically tracked across all recruitment channels, although periodic reposting of the survey link occurred through professional networks during the data collection period.

No incentives were offered for participation. Given the convenience sampling approach and the inability to determine a response denominator, the sample may not be representative of all family physicians in Istanbul. In particular, physicians with greater interest in smoking cessation or e-cigarettes may have been more likely to participate, introducing potential selection bias. These factors should be considered when interpreting the findings.

An explanatory statement was available for participants to read before deciding to proceed with the survey. Once participants began the survey, it was assumed that informed consent was attained. Participation was voluntary, the survey was anonymous and participants were able to exit at any stage of the survey. All procedures were conducted in accordance with Institutional ethical guidelines and approvals.

### Survey and measures

The survey was created in Qualtrics for electronic distribution. Items were adapted from a previously developed questionnaire among Australian general practitioners [26] with modifications to reflect the regulatory and clinical context in Türkiye.

Survey questions were translated from English to Turkish by a bilingual author (MS), and the accuracy of translation reviewed by a second bilingual author (AEO). The translated version was pilot tested with a small number of family physicians (*n* = 5) affiliated with Istanbul University to ensure clarity, comprehension and face validity. No amendments were necessary.

During adaptation of the survey instrument, items related to subjective norms were removed. In the current regulatory and clinical context in Türkiye, recommending e-cigarettes is not an established or widely endorsed practice. As such, items assessing perceived social norms were not expected to capture meaningful variation among respondents and were therefore excluded from the final instrument.

The final questionnaire consisted of 17 items including socio-demographic characteristics, knowledge of e-cigarettes, attitudes toward their safety and effectiveness, perceived behavioural control (confidence in discussing e-cigarettes) and recommendation practices. Survey items were originally drawn from previously published literature [26–33].

**Knowledge** of e-cigarettes was assessed using six items covering key aspects of device characteristics and composition. These included questions on confidence talking to patients about e-cigarettes, whether e-cigarettes contain tobacco or involve combustion, the safety and regulatory status of e-liquid ingredients, device operating characteristics (such as temperature), the presence of quality certification, and whether nicotine-free e-cigarette products exist. Each correct response was assigned 1 point and summed, resulting in a total knowledge score ranging from 0 to 6 points, with higher scores indicating greater knowledge.

**Attitudes** towards e-cigarettes were assessed using items evaluating beliefs about their safety, effectiveness as smoking cessation aids, and potential health risks.

**Perceived behavioural control** was assessed through self-reported confidence in discussing e-cigarettes with patients, answering patient questions, and providing advice.

**Recommendation practices and intentions** were assessed using three binary (Yes/No) items examining willingness to recommend e-cigarettes in different clinical scenarios, including for patients who refused pharmacotherapy or had failed to quit using other methods. The primary outcome measure for the present analysis was willingness to recommend e-cigarettes to patients who had failed to quit using other methods.

The survey design was informed by the Theory of Planned Behaviour (TPB) [25], with measures reflecting attitudes and perceived behavioural control as key determinants of physicians’ recommendation intentions within the study context.

### Statistical analysis

Data were analysed using SPSS Version 29 (IBM SPSS Statistics for Windows, Armonk, NY, USA).

Descriptive statistics were used to summarise participant characteristics and survey responses. Categorical variables were presented as frequencies and percentages, and continuous variables as means and standard deviations (SD).

Bivariate analyses were conducted to explore associations between participant characteristics and key variables. Chi-square tests were used for categorical variables, and independent samples t-tests or one-way analysis of variance (ANOVA) were used for continuous variables.

The primary outcome of interest was family physicians’ willingness to recommend e-cigarettes to patients who had failed to quit using other smoking cessation methods.

To examine factors associated with this outcome, binary logistic regression analyses were performed. Independent variables were selected based on theoretical relevance to the TPB and prior literature, and included measures of:

- Knowledge (total knowledge score).
- Attitudes towards e-cigarettes (e.g., perceived effectiveness and safety).
- Perceived behavioural control (confidence in discussing e-cigarettes with patients).
- Socio-demographic characteristics (age, sex, years of practice, and medical training background).

Univariate logistic regression analyses were initially conducted, followed by multivariable logistic regression models to estimate adjusted associations. Adjusted odds ratios (aORs) with 95% confidence intervals (CIs) were reported.

Missing data were handled using complete-case analysis, with the number of observations included in each model reported. No imputation of missing data was performed. Statistical significance was defined as a two-sided p-value of < 0.05.

## Results

A total of 243 participants completed the survey of fairly equal distribution amongst males (*n* = 116, 47.7%) and females (*n* = 127, 52.3%). Participant characteristics are presented in Table 1. The majority of participants were aged between 30 and 39 years (*n* = 68, 29.1%) and most of the participants practiced in the district of Sultanbeyli (*n* = 34, 14.0%), Bahçelievler (*n* = 23, 9.5%) or Silivri (*n* = 20, 8.2%). Just over half of the participants had clinical practice experience between 0 and 10 years (*n* = 124, 52.1%) and almost all participants obtained their medical degree from Türkiye (*n* = 228, 93.8%). Just over half of respondents reported never smoking (*n* = 136, 56.0%) but 28.8% (*n* = 70) reported currently smoking. Almost all respondents had never used e-cigarettes (*n* = 207, 85.2%).

Most participants believed it was their responsibility to aid patients in getting the correct help to quit smoking (*n* = 159/217, 73.3%) and that current medications are effective in helping patients quit smoking (*n* = 171/217, 78.8%). Most disagreed that e-cigarettes were suitable as smoking cessation aids (*n* = 169/217, 77.9%) or safer than regular cigarettes (*n* = 165/214, 77.1%). Respondents were concerned e-cigarettes were a gateway to smoking (*n* = 153/217, 70.5%), addictive (*n* = 166/217, 76.5%) and harmful to the health of the user (*n* = 162/215, 75.3%). Over half stated they were not confident in their level of knowledge about e-cigarettes (*n* = 126/200, 63%), or their ability to answer patient questions (*n* = 119/200, 59.5%) and talk to patients about e-cigarettes (*n* = 121/199, 60.8%). More than half stated they would not recommend e-cigarettes to patients that refuse to take alternative treatments (*n* = 121/197, 61.4%) or to patients that have failed to quit using other methods (*n* = 118/197, 59.9%).

**Table 1:** Demographic characteristics of respondents

		Total *N* = 243 (%)
Gender	Male	116 (47.7)
	Female	127 (52.3)
Age*	20–29	36 (15.4)
	30–39	68 (29.1)
	40–49	62 (26.5)
	50–59	58 (24.8)
	60+	10 (4.3)
District of clinic^	Ataşehir	13 (5.3)
	Bahçelievler	23 (9.5)
	Bakırköy	3 (1.2)
	Bayrampasa	2 (0.8)
	Beşiktaş	2 (0.8)
	Beykoz	10 (4.1)
	Beylikdüzü	1 (0.4)
	Beyoğlu	2 (0.8)
	Büyükçekmece	1 (0.4)
	Çekmeköy	15 (6.2)
	Esenler	2 (0.8)
	Esenyurt	15 (6.2)
	Eyüpsultan	9 (3.7)
	Fatih	4 (1.6)
	Gaziosmanpaşa	2 (0.8)
	Güngören	1 (0.4)
	İstanbul	4 (1.6)
	Kadıköy	5 (2.1)
	Küçükçekmece	6 (2.5)
	Maltepe	3 (1.2)
	Pendik	2 (0.8)
	Sancaktepe	8 (3.3)
	Sarıyer	1 (0.4)
	Şile	1 (0.4)
	Silivri	20 (8.2)
	Şişli	9 (3.7)
	Sultanbeyli	34 (14.0)
	Sultangazi	3 (1.2)
	Tuzla	1 (0.4)
	Ümraniye	11 (4.5)
	Üsküdar	17 (7.0)
	Zeytinburnu	8 (3.3)
Years of practice^	0–10	124 (52.1)
	11–20	102 (42.9)
	21–30	8 (3.4)
	30+	4 (1.7)
Medical degree obtained^#^	Türkiye	228 (93.8)
	International	14 (5.8)

*n = 9 (3.7%) of participants did not complete this question

^n=5 (2.1%) of participants did not complete this question

^#^n=1 (0.4%) of participants did not complete this question

Less than half of participants (*n* = 77, 39.1%) indicated they would be willing to recommend e-cigarettes to patients for smoking cessation who had failed to quit with other methods. This was reflected equally in both male (*n* = 38, 40.0%) and female (*n* = 39, 38.2%) respondents. Over half of family physicians in all age groups and years of clinical practice were reluctant to support e-cigarettes as an alternative smoking cessation treatment (Table 2) and stated they would not recommend e-cigarettes to patients that refuse to take alternative treatments (*n* = 121, 61.4%) or, to patients that have failed to quit using other methods (*n* = 118, 59.9%).

**Table 2:** Demographics and recommendation of e-cigarettes as smoking cessation aids

If there was a legal regulation regarding the use of e-cigarettes to quit smoking in Türkiye, would you consider recommending e-cigarettes to your patients?	Yes	No	Test Statistic	*p*-value
Gender*				
Male	*n* = 38 (40.0%)	*n* = 57 (60.0%)	χ^2^ = 0.64, df = 1	*p* = 0.800
Female	*n* = 39 (38.2%)	*n* = 63 (61.8%)		
**Age^**				
20–29	*n* = 12 (41.4%)	*n* = 17 (58.6%)	χ^2^ = 1.861, df = 4	*p* = 0.761
30–39	*n* = 22 (40.7%)	*n* = 32 (59.3%)		
40–49	*n* = 21 (44.7%)	*n* = 26 (55.3%)		
50–59	*n* = 17 (32.1%)	*n* = 36 (67.9%)		
60+	*n* = 3 (42.9%)	*n* = 4 (57.1%)		
**Years of Practice** ^**#**^				
0–10	*n* = 36 (35.6%)	*n* = 65 (64.4%)	χ^2^ = 1.512, df = 3	*p* = 0.680
11–20	*n* = 34 (42.0%)	*n* = 47 (58.0%)		
21–30	*n* = 4 (50.0%)	*n* = 4 (50.0%)		
30+	*n* = 1 (25.0%)	*n* = 3 (75.0%)		
Training*				
Türkiye	*n* = 72 (38.7%)	*n* = 114 (61.3%)	χ^2^ = 0.198, df = 1	*p* = 0.656
International	*n* = 5 (45.5%)	*n* = 6 (54.5%)		

* *n* = 46 (18.9%) of participants did not answer this question

^n=53 (21.8%) of participants did not answer this question

#n=49 (20.2%) of participants did not answer this question

Percentages calculated based on available responses

Male family physicians and family physicians with a medical qualification from Türkiye had more knowledge than female family physicians and those with an international qualification. The total average knowledge score was 1.7 (SD = 1.5) out of 6. Family physicians displayed poor knowledge across all six questions and were asked if they would consider recommending e-cigarettes to their patients if the legal regulation in Türkiye permitted the use of e-cigarettes. The majority of family physicians indicated they would not consider recommending e-cigarettes (*n* = 119) or using e-cigarettes (*n* = 134) as an alternative to existing treatments for smoking cessation. Family physicians were also asked about their confidence levels when talking to patients about e-cigarettes. Family physicians who reported being very/somewhat confident (*n* = 78) to speak to patients about e-cigarettes tended to have a higher knowledge score than those who were not at all/somewhat not confident (*n* = 85) although this difference did not reach statistical significance (Table 3).

**Table 3:** Knowledge and recommendation of e-cigarettes as smoking cessation aids

If there was a legal regulation regarding the use of e-cigarettes to quit smoking in Türkiye, would you consider recommending e-cigarettes to your patients?	*N*	Mean (SD)	Test Statistic	*p*-value
**Gender**				
Male	*n* = 111	2.0 (1.5)	T(226) = 3.2	*p* = < 0.001
Female	*n* = 117	1.4 (1.4)		
**Age**				
20–29	*n* = 36	1.8 (1.4)	F = 0.8	*p* = 0.536
30–39	*n* = 65	1.7 (1.5)		
40–49	*n* = 54	1.5 (1.5)		
50–59	*n* = 58	1.7 (1.5)		
60+	*n* = 8	2.4 (1.4)		
**Years of practice**				
0–10	*n* = 118	1.6 (1.4)	F = 0.7	*p* = 0.582
11–20	*n* = 93	1.8 (1.5)		
21–30	*n* = 8	2.1 (1.7)		
30+	*n* = 4	1.0 (2.0)		
**Medical degree obtained**				
Turkish graduate	*n* = 213	1.7 (1.5)	T(225) = 1.8	*p* = 0.037
International graduate	*n* = 14	1.0 (1.0)		
**Confidence talking to patients about e-cigarettes**				
*Not at all/somewhat not*	*n* = 85	1.4 (1.3)	F = 2.0	*p* = 0.130
*Neither/nor*	*n* = 35	1.9 (1.4)		
*Very/somewhat*	*n* = 78	1.8 (1.6)		
**I WOULD NOT recommend using e-cigarettes or vaping**				
I would not recommend	*n* = 134	1.6 (1.5)	T(226) = 0.816	*p* = 0.208
No response	*n* = 94	1.8 (1.4)		
**Would you consider recommending e-cigarettes to your patients?**				
Yes	*n* = 77	1.8 (1.4)	T(194) = 1.1	*p* = 0.132
No	*n* = 119	1.6 (1.5)		

Family physicians’ attitudes towards the use of e-cigarettes as smoking cessation aids were negative. The majority did not recommend e-cigarettes to their patients (in line with current guidelines) and most believed e-cigarettes should not be regarded as a type of smoking cessation aid (*n* = 82, 68.3%), and that they do not help patients quit smoking (*n* = 79, 66.4%), or lower the risk of tobacco related diseases (*n* = 62, 51.2%). They did not consider e-cigarettes as safer (*n* = 78, 65.5%) than regular cigarettes or more effective than other smoking cessation treatments (*n* = 84, 71.2%). Family physicians who were not willing to recommend e-cigarettes, agreed they are carcinogenic (*n* = 88, 73.9%) and a gateway to smoking (*n* = 95, 79.2%) (Table 4).

**Table 4:** Attitudes and recommendation of e-cigarettes as smoking cessation aids

If there was a legal regulation regarding the use of e-cigarettes to quit smoking in Türkiye, would you consider recommending e-cigarettes to your patients?	Yes	No	Test Statistic	*p*-value
E-cigs are a gateway to smoking*				
Strongly/somewhat disagree	*n* = 18 (23.4%)	*n* = 15 (12.5%)	χ^2^ = 15.3, df = 2	*p* = < 0.001
*Neither/nor*	*n* = 18 (23.4%)	*n* = 10 (8.3%)		
*Strongly/somewhat agree*	*n* = 41 (53.2%)	*n* = 95 (79.2%)		
E-cigs can be addictive*				
*Strongly/somewhat disagree*	*n* = 11 (14.3%)	*n* = 7 (5.8%)	χ^2^ = 5.5, df = 2	*p* = 0.064
*Neither/nor*	*n* = 14 (18.2%)	*n* = 16 (13.3%)		
*Strongly/somewhat agree*	*n* = 52 (67.5%)	*n* = 97 (80.8%)		
E-cigs can be regarded as a type of smoking cessation aid*				
*Strongly/somewhat disagree*	*n* = 34 (44.2%)	*n* = 82 (68.3%)	χ^2^ = 16.8, df = 2	p = < 0.001
*Neither/nor*	*n* = 14 (18.2%)	*n* = 22 (18.3%)		
*Strongly/somewhat agree*	*n* = 29 (37.7%)	*n* = 16 (13.3%)		
**E-cigs can decrease the number of cigarettes smoked^**				
*Strongly/somewhat disagree*	*n* = 16 (21.1%)	*n* = 50 (41.7%)	χ^2^ = 24.3, df = 2	p = < 0.001
*Neither/nor*	*n* = 8 (10.5%)	*n* = 31 (25.8%)		
*Strongly/somewhat agree*	*n* = 52 (68.4%)	*n* = 39 (32.5%)		
**E-cigs can lower the risk of tobacco-related diseases#**				
*Strongly/somewhat disagree*	*n* = 14 (18.4%)	*n* = 62 (52.1%)	χ^2^ = 34.7, df = 2	p = < 0.001
*Neither/nor*	*n* = 11 (14.5%)	*n* = 27 (22.7%)		
*Strongly/somewhat agree*	*n* = 51 (67.1%)	*n* = 30 (25.2%)		
**E-cigs can help patients quit smoking#**				
*Strongly/somewhat disagree*	*n* = 21 (27.6%)	*n* = 79 (66.4%)	χ^2^ = 44.1, df = 2	p = < 0.001
*Neither/nor*	*n* = 10 (13.2%)	*n* = 23 (19.3%)		
*Strongly/somewhat agree*	*n* = 45 (59.2%)	*n* = 17 (14.3%)		
**E-cigs are safer than regular cigarettes+**				
*Strongly/somewhat disagree*	*n* = 22 (29.3%)	*n* = 78 (65.5%)	χ^2^ = 24.2, df = 2	p = < 0.001
*Neither/nor*	*n* = 26 (34.7%)	*n* = 20 (16.8%)		
*Strongly/somewhat agree*	*n* = 27 (36.0%)	*n* = 21 (17.6%)		
**E-cigs have adverse health effects#**				
*Strongly/somewhat disagree*	*n* = 5 (6.6%)	*n* = 3 (2.5%)	χ^2^ = 2.9, df = 2	*p* = 0.236
*Neither/nor*	*n* = 15 (19.7%)	*n* = 18 (15.1%)		
*Strongly/somewhat agree*	*n* = 56 (73.7%)	*n* = 98 (82.4%)		
**E-cigs are less harmful than regular cigarettes+**				
*Strongly/somewhat disagree*	*n* = 16 (21.3%)	*n* = 58 (48.7%)	χ^2^ = 20.0, df = 2	p = < 0.001
*Neither/nor*	*n* = 18 (24.0%)	*n* = 31 (26.1%)		
*Strongly/somewhat agree*	*n* = 41 (54.7%)	*n* = 30 (25.2%)		
**E-cig use is harmful for the health of the user#**				
*Strongly/somewhat disagree*	*n* = 9 (11.8%)	*n* = 10 (8.4%)	χ^2^ = 3.3, df = 2	*p* = 0.188
*Neither/nor*	*n* = 15 (19.7%)	*n* = 14 (11.8%)		
*Strongly/somewhat agree*	*n* = 52 (68.4%)	*n* = 95 (79.8%)		
**E-cigs are carcinogenic#**				
*Strongly/somewhat disagree*	*n* = 15 (19.7%)	*n* = 4 (3.4%)	χ^2^ = 16.4, df = 2	p = < 0.001
*Neither/nor*	*n* = 21 (27.6%)	*n* = 27 (22.7%)		
*Strongly/somewhat agree*	*n* = 40 (52.6%)	*n* = 88 (73.9%)		
**E-cigs are more effective than other smoking cessation aids+**				
*Strongly/somewhat disagree*	*n* = 39 (51.3%)	*n* = 84 (71.2%)	χ^2^ = 7.9, df = 2	*p* = 0.019
*Neither/nor*	*n* = 18 (23.7%)	*n* = 17 (14.4%)		
*Strongly/somewhat agree*	*n* = 19 (25.0%)	*n* = 17 (14.4%)		

*n = 46 (18.9%) of participants did not answer these questions

^n=47 (19.3%) of participants did not answer these questions

#n=48 (19.8%) of participants did not answer these questions

+n=49 (20.2%) of participants did not answer these questions

Percentages calculated based on available responses

In regards to confidence discussing e-cigarettes, family physicians that were most confident in their level of knowledge about e-cigarettes were least likely to believe they had use in smoking cessation (*n* = 50, 41.5%). Similarly, family physicians that were confident in their abilities to talk to patients about e-cigarettes were unlikely to recommend them to their patients to cease smoking (*n* = 54, 45%) (Table 5).

**Table 5:** Behavioural control and recommendation of e-cigarettes

If there was a legal regulation regarding the use of e-cigarettes to quit smoking in Türkiye, would you consider recommending e- cigarettes to your patients?	Yes	No	Test Statistic	*p*-value
**Confidence with Level of knowledge about e-cigarettes^**				
*Not at all/somewhat not*	*n* = 40 (51.9%)	*n* = 41 (34.2%)	χ^2^ = 6.1, df = 2	*p* = 0.046
*Neither/nor*	*n* = 14 (18.2%)	*n* = 29 (24.2%)		
*Very/somewhat*	*n* = 23 (29.9%)	*n* = 50 (41.7%)		
**Ability to answer questions from patients about e-cigarettes^**				
*Not at all/somewhat not*	*n* = 40 (51.9%)	*n* = 44 (36.7%)	χ^2^ = 4.6, df = 2	*p* = 0.100
*Neither/nor*	*n* = 12 (15.6%)	*n* = 22 (18.3%)		
*Very/somewhat*	*n* = 25 (32.5%)	*n* = 54 (45.0%)		
**Ability to talk to patients about e-cigarettes#**				
*Not at all/somewhat not*	*n* = 44 (57.9%)	*n* = 41 (43.2%)	χ^2^ = 10.7, df = 2	*p* = 0.005
*Neither/nor*	*n* = 10 (13.2%)	*n* = 25 (20.8%)		
*Very/somewhat*	*n* = 22 (28.9%)	*n* = 54 (45.0%)		

^n=46 (18.9%) of participants did not answer this question

#n=47 (19.3%) of participants did not answer this question

Percentages calculated based on available responses

The primary outcome of interest was family physicians’ willingness to recommend e-cigarettes as a substitute to people who smoke and who had failed to quit with other methods. A generalized ordered logistic regression model was used to examine factors associated with this outcome (Table 6) adjusting for age and sex. In multivariable analysis, family physicians with lower knowledge scores (< 3) had significantly lower odds of recommending e-cigarettes compared with those with higher knowledge scores (aOR = 0.48, 95%CI: 0.25–0.90; *p* = 0.024). Compared with physicians who were neutral, those who agreed e-cigarettes can help patients quit smoking had significantly higher odds of recommending them (aOR = 4.17, 95%CI: 1.67–10.14; *p* = 0.002). Physicians who disagreed e-cigarettes can help patients quit smoking showed a trend towards lower odds of recommending e-cigarettes, however this association was not statistically significant at the *p* < 0.05 level. No significant associations were observed for other variables included in the model, including age, sex, years in practice, and medical training background (Table 6).

**Table 6:** Regression estimates for family physicians (*N* = 243)

Variables	Would you recommend electronic cigarettes to smokers who failed to quit with other methods? (*N* = 243)	
	Univariate OR 95% CI; *p* value	Multivariate aOR* 95% CI; *p* value
**Years of GP practice** < 10 years (*n* = 102)		
	1.20(0.34, 4.27);0.771	0.67(0.14, 3.10);0.613
10–20 years (*n* = 124)	1.52 (0.42, 5.46);0.520	1.21(0.31, 4.60); 0.776
**Reference**: >20years (*n* = 12)	----	----
**Medical Degree obtained with a Turkish University**		
**Reference**: Türkiye (*n* = 228)	1.84 (0.43, 7.16);0.473	1.94(0.49, 7.62); 0.340
International (*n* = 14)	----	----
**Knowledge** Total Knowledge Score		
Low (*n* = 121)	0.57(0.32, 1.02);0.060	**0.48(0.25**,** 0.90);0.024**
**Reference**: High (*n* = 107)	----	----
**E-cigs can help patients quit smoking**		
Strongly/somewhat disagree (*n* = 114)	**0.40(0.17**,** 0.95);0.039**	**0.43(0.15**,** 1.03);0.059**
Strongly/somewhat agree (*n* = 64)	**4.07(1.66**,** 9.95);0.002**	**4.17(1.67**,** 10.14);0.002**
**Reference**: neither/nor (*n* = 37)	----	----
**Ability to talk to patients about e-cigs**		
Not at all/somewhat not confident (*n* = 86)	1.53(0.69, 3.41);0.293	0.51(0.21, 1.21);0.131
Very/somewhat confident (*n* = 78)	0.57(0.24, 1.33);0.195	1.35 (0.59, 3.07);0.469
**Reference**: neither/nor (*n* = 35)	----	----

Note: OR= Odds Ratio; aOR= adjusted OR; 95% CI = 95% Confidence intervals; Total Knowledge Score grouped as low if the score is below median average (< 3) and score more than or equal to 3 considered as having high Total Knowledge Score. *Model adjusted for the age, sex, years in GP practice and medical degree

## Discussion

This study examined family physicians’ knowledge, attitudes, and perceived behavioural control regarding e-cigarettes, and how these factors were associated with their willingness to recommend e-cigarettes for smoking cessation. Overall, respondents were reluctant to recommend e-cigarettes to their patients as a method for smoking cessation. Male family physicians scored higher than female family physicians in knowledge of e-cigarettes, and family physicians with more negative attitudes towards e-cigarettes were less likely to endorse their use for smoking cessation. Participants demonstrated poor knowledge and low confidence in discussing e-cigarettes and held predominantly negative attitudes toward there use in smoking cessation. Consistent with this, the majority were reluctant to recommend e-cigarettes in clinical scenarios.

Importantly, the multivariable analysis provided further insight into the factors underlying these intentions. Physicians’ attitudes, particularly beliefs about the effectiveness of e-cigarettes for smoking cessation, were strongly associated with willingness to recommend them. Knowledge also contributed to recommendation intentions when categorised as high versus low, whereas perceived behavioural control (confidence), was not independently associated with recommendation behaviour in adjusted models.

These findings are broadly consistent with previous research conducted with Turkish family physicians in Istanbul, including by our group [23] and by Tanriover and colleagues [16], which reported low levels of e-cigarette recommendation and limited confidence in smoking cessation counselling. However, while these prior studies have predominantly described levels of awareness, beliefs, and practices, the present study extends this literature by examining how these factors relate to each other within a theory-informed framework [25]. In particular, findings suggest that physicians’ intentions are more strongly associated with their beliefs about the effectiveness and safety of e-cigarettes, than with their confidence discussing them. This provides additional insight into the mechanisms that may underpin clinical-decision making in this context, beyond descriptive associations.

The family physicians in this study expressed poor knowledge about e-cigarettes, believed e-cigarettes to be a gateway to smoking and other tobacco products, opposed to recommending e-cigarettes as a smoking cessation alternative, and did not feel comfortable or confident enough to have discussions with their patients about e-cigarettes for smoking cessation [23]. Similarly, Tanriover et al. (2022) found that the vast majority of family physicians did not recommend e-cigarettes to their patients for smoking cessation [16] and this was echoed in other international studies by Christen et al. (2025), reporting that 63% of healthcare professionals in Switzerland were reluctant to recommend e-cigarettes as smoking cessation aids [34]. Furthermore, a study conducted by Arslan et al. (2020) evaluated the opinions of family physicians in Türkiye on some tobacco products including e-cigarettes [35]. Their study found that approximately 85% of family physicians were aware of e-cigarettes, considered them to be addictive, and were aware their use was prohibited in closed public areas [35]. While our findings are broadly consistent with previous studies, the present study adds further insight by examining how knowledge, attitudes and perceived behavioural control relate to physicians’ recommendation intentions.

Both male and female family physicians in our sample regardless of their age or years of experience, were generally not willing to recommend e-cigarettes to their patients for smoking cessation. Similar to our findings, physicians in other countries have reported reluctance to recommend e-cigarettes as an alternative to smoking or as a smoking cessation aid [27, 30, 36]. In Australia, where a prescription model for e-cigarettes was introduced in 2021, general practitioners showed limited support for their use compared with existing smoking cessation therapies, citing concerns about safety, efficacy, and potential for increased nicotine addiction in the community [26, 37]. This contrasts with approaches in countries such as the UK and New Zealand, where e-cigarettes are more widely integrated into tobacco harm reduction strategies [38–40].

Our study showed that male family physicians had higher knowledge scores than female family physicians; however, this did not translate into a greater likelihood of recommending e-cigarettes. More than half of family physicians, regardless of knowledge level, were not open to recommending e-cigarettes to their patients, consistent with current guidelines and regulations in Türkiye. Overall, Turkish family physicians in Istanbul demonstrated relatively low levels of knowledge about e-cigarettes, which is consistent with other international literature [41].

Encouragingly, those family physicians in Istanbul who reported greater confidence in their abilities to talk to patients about e-cigarettes also had higher knowledge score. This suggests a need for further training and education for family physicians on e-cigarettes, to increase their confidence in discussing these products with their patients and providing evidence-based advice when patients enquire about their use to quit smoking. These findings suggest that improving knowledge may be important for shaping physicians’ beliefs about e-cigarettes and supporting informed discussions with patients.

Family physicians in this sample held predominately negative attitudes towards the efficacy of e-cigarette use for smoking cessation and would not recommend them to patients who want to quit smoking. These family physicians had strong beliefs about e-cigarettes, that e-cigarettes may act as a gateway to smoking, are addictive, and potentially harmful, consistent with other international studies [16, 24, 26, 27, 32, 35, 36, 42–44]. Additionally, over half of family physicians disagreed that e-cigarettes could function as a smoking cessation aid, help patients quit smoking, or are safer or more effective than other smoking cessation methods. In line with regression findings, these attitudes, particularly beliefs about effectiveness, appear to play an important role in shaping physicians’ willingness to recommend e-cigarettes. Qualitative research with Turkish family physicians in Istanbul suggests these beliefs may reflect uncertainty and a lack of confidence in the evidence base, contributing to limited trust in the safety and efficacy of e-cigarettes as smoking cessation aids [23].

### Strengths and limitations

This study examined Turkish family physicians’ knowledge, attitudes, and beliefs about e-cigarettes and their use in smoking cessation. A strength of this research is the relatively large sample size and the use of survey items drawn from published literature and informed by the Theory of Planned Behaviour, allowing comparison with international studies. The inclusion of multivariable analyses also enabled examination of factors associated with recommendation intentions.

However, several limitations should be considered. The study utilised a convenience sampling approach, and the absence of a defined sampling frame and response rate limits the ability to assess representativeness and introduces the potential for selection bias. Physicians with a particular interest in smoking cessation or e-cigarettes may have been more likely to participate. In addition, the sample was limited to Istanbul, the major metropolitan centre in Türkiye, and the findings may not be generalised to all family physicians in other regions of the country.

The survey relied on self-reported data and was subject to response bias, and not all family physicians completed all survey items, resulting in missing data in some analyses. Complete-case analysis was used, and no imputation was performed. Finally, although the study was informed by the Theory of Planned Behaviour, subjective norms were not assessed due to the limited expected variability within the study context.

## Conclusion

There are a limited number of studies exploring Turkish family physicians’ perceptions of e-cigarettes and their potential role in smoking cessation. Our results show that family physicians in Istanbul were generally reluctant to recommend e-cigarettes for smoking cessation, and this may reflect both the regulatory context at the time of the survey and prevailing clinical beliefs. Knowledge about e-cigarettes was poor, and many family physicians reported low confidence in discussing e-cigarettes with their patients. The findings suggest that improving access to trusted, evidence-based information about e-cigarettes may support family physicians in responding to patient enquiries and engaging in informed discussions about smoking cessation options.

## Supplementary Information

Below is the link to the electronic supplementary material.

Supplementary Material 1

## Data Availability

The authors confirm that the data supporting the findings of this study are available within the article (and/or) its supplementary materials.

## Abbreviations

E-cigarettes: Electronic Cigarettes
FCTC: Framework Convention on Tobacco Control
FPs: Family Physicians
TPB: Theory of Planned Behaviour
