# Supplementary material for: Perspectives of Turkish family physicians in Istanbul about e-cigarettes as smoking cessation aids: a cross-sectional survey study
Source: Addict Sci Clin Pract. 2026 Jul 27;21:57. doi: 10.1186/s13722-026-00707-w (PMC13418429; doi:10.1186/s13722-026-00707-w)
Supplement: Supplementary file 1 — Supplementary Material 1 [file 13722_2026_707_MOESM1_ESM.pdf]

Supplementary File S1. Survey in English

Turkish FPs perceptions of e-cigarettes as a smoking cessation aid

Q1. What is your age (years)?

____

Q2. What is your gender?

- Male
- Female
- Non-binary / third gender
- Other
- Prefer not to say

Q3. What is the name of the suburb that your clinic is located in? (list multiple if applicable)

____

Q4. How many years have you worked as a GP?

____

Q5. In which country did you receive your medical training?

____

Q6. What other qualifications do you hold (please specify)?

____

Q7. Do you smoke cigarettes, pipes or other tobacco products?

- Daily
- At least once a week
- Less than weekly
- Not at all but I have been a regular smoker in the past
- Not at all and I have never been a regular smoker

Q8. Do you vape or use e-cigarettes?

- Daily
- At least once a week
- Less than weekly
- Not at all but I have been a regular e-cigarette user in the past
- Not at all and I have never been a regular e-cigarette user

Q9. In your opinion which of the following are correct concerning e-cigarettes in Türkiye?

	Yes	Unsure	No
They contain tobacco	●	●	●
There is combustion	●	●	●
E-liquid ingredients are approved for inhalation	●	●	●
Working temperature is lower than in tobacco cigarettes	●	●	●
They have official quality certificates	●	●	●
There are e-cigarettes without nicotine	●	●	●

Q10. The following questions ask about your knowledge, beliefs and the safety and efficacy of e-cigarettes in the context of smoking cessation. Please indicate your response for each question.

	Strongly disagree	Somewhat disagree	Neither agree nor disagree	Somewhat agree	Strongly agree
It is part of my responsibility as a GP to make sure that my patients get the help they need to quit smoking					
GP advice to quit is effective in helping patients quit smoking					
Current medications such as varenicline (champix)/bupropion (zyban)/nicotine gum/patches are effective in helping patients quit smoking					
E-cigarettes are a gateway to smoking					
E-cigarettes can be addictive					
E-cigarettes can be regarded as a type of smoking cessation aid					
E-cigarettes can decrease the number of cigarettes smoked					

E-cigarettes can lower the risk of tobacco-related diseases	●	●	●	●	●
E-cigarettes can help patients quit smoking	●	●	●	●	●
E-cigarettes are safer than regular cigarettes	●	●	●	●	●
E-cigarettes have adverse health effects	●	●	●	●	●
E-cigarettes are less harmful than regular cigarettes	●	●	●	●	●
E-cigarette use is harmful for the health of the user	●	●	●	●	●
E-cigarette aerosol is harmful for people in the vicinity of the users	●	●	●	●	●
E-cigarettes are carcinogenic	●	●	●	●	●
E-cigarettes are more effective than other smoking cessation treatments	●	●	●	●	●

Q11. Where do you receive e-cigarette information from? (Select all that apply)

- ☐ Scientific literature
- ☐ Professional Organisations (please specify) ________________________________
- ☐ Government reports/websites
- ☐ Non-government organisations
- ☐ Media (TV, radio, newspaper, internet)
- ☐ Social media
- ☐ Patients
- ☐ Health care colleagues
- ☐ E-cigarette companies/retailers
- ☐ None of these/have not received information about e-cigarettes
- ☐ Other (please specify) ____

Q12. Please indicate your level of confidence for each question.

	Not at all confident	Somewhat not confident	Neither confident nor not confident	Somewhat confident	Very confident
Your ability to talk with your patients about their smoking?	●	●	●	●	●
Your knowledge about medications used to help patients quit smoking?	●	●	●	●	●
Your ability to help your patients stop smoking?	●	●	●	●	●
Your level of knowledge about e-cigarettes	●	●	●	●	●
Your ability to answer questions from patients about e-cigarettes	●	●	●	●	●
Your ability to talk to your patients about e-cigarettes for smoking cessation	●	●	●	●	●

Q13. Would your advice to patients about using e-cigarettes, or vaping, include any of the following (please tick all that apply)

- ☐ I would say that some patients find using e-cigarettes, or vaping, helpful to stop smoking tobacco
- ☐ I would only recommend using e-cigarettes, or vaping, as a secondary approach to stop smoking tobacco (i.e. if all other medicinal therapies have failed)
- ☐ I would let the patient choose whether or not they want to use e-cigarettes, or vape, to stop smoking tobacco
- ☐ I would recommend using e-cigarettes, or vaping, to my patients as a first line therapy for stopping smoking tobacco
- ☐ I would recommend using e-cigarettes, or vaping, to tobacco smokers who do not intend to quit or who have declined the offer of help
- ☐ Other advice (please specify) ____
- ☐ I would not offer any advice about using e-cigarettes, or vaping to patients
- ☐ I do not recommend using e-cigarettes, or vaping, to patients

---

Q14. Some countries have policy and guidelines around e-cigarettes as smoking cessation aids. If Türkiye had regulated a law for the use of e-cigarettes to quit smoking, would you consider recommended e-cigarettes to your patients?

- ○ Yes
- ○ No

Q15. Would you consider recommending e-cigarettes as a substitute to smokers who would refuse to take medications for smoking cessation?

- ○ Yes
- ○ No

Q16. Would you consider recommending e-cigarettes to smokers who failed to quit with other methods?

- Yes
- No

Q17. If there is anything further you would like to share about your experiences or concerns discussing e-cigarettes with your patients please use the text box below.

____

____

____

____

____
